# The impact of β-glucan on the therapeutic outcome of experimental *Trichinella spiralis* infection

**DOI:** 10.1007/s00436-023-07964-7

**Published:** 2023-09-22

**Authors:** Dina I. Elgendy, Ahmad A. Othman, Mohamed M. Eid, Samy I. El-Kowrany, Fersan A. Sallam, Dareen A. Mohamed, Doaa. H. Zineldeen

**Affiliations:** 1 Medical Parasitology Department, Faculty of Medicine, Tanta University, Tanta, Egypt; 2 Pathology Department, Faculty of Medicine, Tanta University, Tanta, Egypt; 3 Medical Biochemistry and Molecular Biology Department, Faculty of Medicine, Tanta University, Tanta, Egypt; 4 College of Medicine, Sulaiman AlRajhi University, 51942 Albukairiyah, Saudi Arabia

**Keywords:** *Trichinella*, Treatment, β-glucan, Albendazole, IL-5, TGF-β, NF-κB

## Abstract

Trichinellosis is a cosmopolitan zoonosis that is caused mainly by *Trichinella spiralis* infection. The human disease ranges from mild to severe and fatality may occur. The treatment of trichinellosis still presents a challenge for physicians. Anti-inflammatory drugs are usually added to antiparasitic agents to alleviate untoward immuno-inflammatory responses and possible tissue damage but they are not without adverse effects. Thus, there is a need for the discovery of safe and effective compounds with anti-inflammatory properties. This study aimed to evaluate the activity of β-glucan during enteral and muscular phases of experimental *T. spiralis* infection as well as its therapeutic potential as an adjuvant to albendazole in treating trichinellosis. For this aim, mice were infected with *T. spiralis* and divided into the following groups: early and late β-glucan treatment, albendazole treatment, and combined treatment groups. Infected mice were subjected to assessment of parasite burden, immunological markers, and histopathological changes in the small intestines and muscles. Immunohistochemical evaluation of NF-κB expression in small intestinal and muscle tissues was carried out in order to investigate the mechanism of action of β-glucan. Interestingly, β-glucan potentiated the efficacy of albendazole as noted by the significant reduction of counts of muscle larvae. The inflammatory responses in the small intestine and skeletal muscles were mitigated with some characteristic qualitative changes. β-glucan also increased the expression of NF-κB in tissues which may account for some of its effects. In conclusion, β-glucan showed a multifaceted beneficial impact on the therapeutic outcome of *Trichinella* infection and can be regarded as a promising adjuvant in the treatment of trichinellosis.

## Introduction

Trichinellosis is a helminthozoonosis that affects people all around the world (Gottstein et al. 2009). According to estimates, up to 11 million people worldwide are infected with *Trichinella* species, and many more are at risk of acquiring the infection (Dupouy-Camet 2000). The main source of infections for humans is *Trichinella spiralis* (*T. spiralis*), and pork that has been undercooked is the main source of infection. Numerous mammalian hosts, including mice, pigs, bears, and humans, are susceptible to the infection (Jasmer 1993). This parasite has two exceptional characteristics that influence the host immune reaction: first, it completes the life cycle in the same host, and second, both the adult worms and the larvae inhabit two different intracellular niches. The adult worms colonize the intestinal epithelium while the larvae reside in the skeletal muscle fibers (Fabre et al. 2009).

The severity of the human disease ranges from moderate to severe, and deaths may supervene. However, as there have been no prospective, controlled clinical trials of treatment for this infection, therapy continues to be difficult and controversial (Watt and Silachamroon 2004). Empirically, the majority of experts advise using corticosteroids in addition to diffusible anthelmintics. The treatment outcomes, however, are not always satisfactory (Dupouy-Camet 2000).

Treatment of trichinellosis is still a dilemma for the physicians. One of the biggest issues is that the timing of administration is crucial to the effectiveness of anthelminthic drugs in treating trichinellosis. In fact, when given early, while adult worms are still in the small intestine, or when larvae are migrating to the muscles, anthelmintics are very effective. In contrast, the majority of patients present at a late stage of the illness, when the skeletal muscle fibers have already been thoroughly colonized by the larvae (Gottstein et al. 2009). Another challenge is the limited bioavailability of the existing anthelmintics due to their low water solubility and diffusibility. Low levels of the medications are therefore attainable around the encapsulated larvae with less than adequate response within a safe therapeutic range (Casulli et al. 2006).

Furthermore, *T. spiralis* infection is characterized by significant inflammatory alterations in the affected tissues. Inadvertently, the death of parasites within the tissues by the action of anthelmintics has the potential to exacerbate the inflammatory reactions, which is particularly harmful in vital organs like the heart or brain. Anti-inflammatory medications are thus regarded as an integral component in the treatment of this infection (Shimoni et al. 2007). Anti-inflammatory agents, whether non-steroidal or steroidal, have a number of adverse effects and contraindications that may limit their usefulness (Barnes 2014; Badri et al. 2016; Oray et al. 2016). Finding new, safe, and effective agents with anti-inflammatory activity is therefore mandatory.

β-glucans are potent immunomodulatory agents that have the power to alter immune responses (Leung et al. 2006). The primary source of β-glucans in nature is fungi, particularly yeast and mushrooms (Wakshull et al. 1999). They are also prepared from plants, such as cereal grains such as barley and oat, and from bacteria and algae (Vetvicka and Yvin 2004; McIntosh et al. 2005). Briefly, β-glucans are found in a wide range of natural sources belonging to various taxonomic groups among prokaryotes and eukaryotes (Volman et al. 2008). It is frequently used as an over-the-counter nutraceutical with a large safety margin.

Neutrophils, B cells, T cells, and natural killer cells could all be stimulated by β-glucans, but macrophages and dendritic cells are generally regarded to be their main target cells (Vetvicka 2011). As they increase the ability of macrophages, neutrophils, and natural killer cells to respond to and attack a variety of pathogens such as viruses, bacteria, fungi, and parasites, they boost the body’s immune system defense against external invaders (Rondanelli et al. 2009). Some studies have shown enhanced efficacy of antiparasitic therapy when β-glucans are added (Dymon and Papir 2004), such as in cases of toxocariasis (Hrckova et al. 2007) and acute toxoplasmosis (Büyükbaba Boral et al. 2012).

This study aimed to evaluate the immunomodulatory activity of β-glucan during enteral and muscular phases of experimental *T. spiralis* infection as well as its potential as an adjuvant to albendazole in the treatment of *Trichinella* infection. We have found that β-glucan has a multifaceted beneficial impact on the therapeutic outcome of *Trichinella* infection and can be considered a promising adjuvant in the treatment of trichinellosis.

## Material and methods

### Parasites and animals

According to Dunn and Wright’s (1985), *T. spiralis* L1 larvae were used to infect mice. A total of 200 larvae were given orally to each animal in a single dose. The *Trichinella* species used in this investigation was genotyped as *T. spiralis* (ISS6158) by the Superior Institute of Health, Rome, Italy’s European Union Reference Laboratory for Parasites. We utilized male Swiss albino mice that were 6–8 weeks old and weighed 25–30 g apiece. Animals were provided by Theodore Bilharz Research Institute (Giza, Egypt) and were thereafter housed and handled in accordance with the institutional and national guidelines.

### Drugs

A commercial preparation of albendazole (Alzental) suspension (Eipico, Egypt) which contains 20 mg/ml was used. The required dose (50 mg/kg body weight/day for 14 successive days) according to Li et al. (2012) was administered orally to each mouse. As regards β-1,3-glucan, a commercial preparation of the drug, yeast-free β-glucan (*Agaricus* mushroom) (Paradise Herbs & Essentials, Inc., USA) which contains 250 mg/capsule was used. The required dose (5 mg/kg body weight/day for 14 successive days) according to Büyükbaba et al. (2012) was administered orally to each mouse after the appropriate dilution with sterile distilled water.

### Experimental design

Mice were divided into six groups: group I (20 mice): uninfected control; group II (25 mice): *T. spiralis*-infected untreated control; group III (20 mice): infected mice that were treated with albendazole starting on the 21st day p.i. for 14 successive days; group IV (25 mice): infected mice that were treated with β-glucan starting on the 1st day p.i. for 14 successive days; group V (20 mice): infected mice that were treated with β-glucan starting on the 21st day p.i. for 14 successive days; and group VI (20 mice): infected mice that were treated with both β-glucan and albendazole starting on the 21st day p.i. for 14 successive days.

Fourteen days post-infection (p.i.), 5 mice from both group II and group IV were euthanized, and their small intestines were taken for assessment of adult worm counts. At 35–37 days p.i., 20 mice from each group were euthanized and subjected to the following: total larval count in muscles, estimation of cytokines in intestinal homogenates and in sera, and histopathological and immunohistochemical study on the small intestine and skeletal muscle samples.

### Parasitological assay

#### Assessment of adult worm counts

Five animals of infected control and early β-glucan treatment groups were euthanized 14 days p.i., and adult worm counts in the small intestines were determined as described by Wakelin and Lloyed (1976).

#### Assessment of total larval counts

Infected mice’s total muscle larval numbers were calculated using the Dunn and Wright (1985) method.

### Histopathological evaluation

After being promptly fixed by immersion in 10% formalin, tissue samples from the small intestine (1 cm from the middle of the jejunum) and skeletal muscles (pieces from the diaphragm and thigh muscles) of the study groups underwent routine histological processing, paraffin embedding, and microtomy. Slides were randomly selected and evaluated blindly and independently by 2 examiners.

#### Differential assessment of cells of the inflammatory infiltrate in the intestine

Based on the number of cells, a semi-quantitative score of five grades was given to each case as follows: ( −): absent, ( +): mild, (+ +): moderate, (+ + +): strong, and (+ +  + +): marked. Each histological section had 20 high-power fields (× 40) examined in order to evaluate the preceding histopathological parameters. The average score was then determined.

#### Image analysis for evaluation of intestinal and skeletal muscle pathology

This was done using an image analyzer (LEICA DFG 290 HD) at the central laboratory of Tanta Faculty of Medicine, Tanta, Egypt. For evaluation of the previous histopathological parameters, an examination of 20 high-power fields (× 40) in each histological section was done, and the average score was calculated. Moreover, a blind assessment was carried out where the specimens were randomized and coded before examination by 2 pathologists.

### Assessment of immunological parameters

#### Preparation of samples for ELISA measurements

Utilizing serum separator tubes, samples were centrifuged for 15 min at 1000 × g after clotting for 2 h at room temperature or overnight at 4°C. The serum was taken out and kept at − 70°C.

#### Small intestinal homogenates

A 100 mg washed clean piece of the mid-intestinal region was homogenized in 1 ml of PBS, refrigerated overnight at − 20°C, and then rinsed with PBS. The homogenates were centrifuged for 5 min at 5000 g, 2‒8°C, following two freeze–thaw cycles that were used to rupture the cell membranes. The fluid from the supernatant was taken out and kept at − 70°C. Before the assay, the samples were centrifuged once more after thawing.

#### Estimation of levels of interleukin-5 (IL-5)

This was done in small intestinal homogenates and in serum using platinum ELISA kits for quantitative detection of mouse IL-5 (eBioscience, CA, USA).

#### Estimation of levels of transforming growth factor-β (TGF-β)

This was done in small intestinal homogenates and in serum using platinum ELISA kits for quantitative detection of mouse TGF-β (eBioscience, CA, USA).

### Immunohistochemistry for assessment of NF-κB expression

Immunohistochemistry was carried out for the demonstration of NF-κB -expressing cells using rabbit anti-phospho-NF-κB p65 ser276 antibody (NF kappa B p65) (henceforth pp65, Cell Signaling, Danvers, MA). After peroxidase blockage and microwave antigen retrieval (using citrate buffer at pH 6.0), 3–5 μm tissue sections were incubated with the primary antibody overnight at 4°C. For the negative control, the primary antibody was replaced with PBS. Rabbit anti-mouse horseradish peroxidase-conjugated secondary antibody was added followed by incubation for 40 min at room temperature. The color was developed using diaminobenzidine as a chromogen. Slides were extensively washed with PBS after each step. Finally, they were counter-stained with Mayer’s hematoxylin.

Immunoreactivity of NF-κB appeared as brown cytoplasmic and nuclear staining of varying degrees of intensity in epithelial and inflammatory cells. For negative control, the primary antibody was replaced by PBS. For the estimation of the number of NF-κB positive cells, image analysis was performed on immuno-stained sections to measure the number of NF-κB-positive cells whether nuclear or cytoplasmic. Ten random non-overlapping fields in each slide were examined and digitally imaged at a magnification of × 400 (Ashour et al. 2015).

### Statistical analysis

Quantitative data were presented as mean ± standard deviation. The probability of significant differences among groups was determined by the Kruskal–Wallis test, a one-way ANOVA test followed by Tukey’s post-hoc test. Differences were considered significant when *P*-value was < 0.05. The statistical analyses were processed according to the conventional procedures using Statistical Package of Social Sciences (SPSS Inc., Chicago, IL, USA) software for Windows, version 10.0.

## Results

### Parasite burden in the small intestines and muscles

There was no statistically significant difference (*P* > 0.05) in adult worm counts in the small intestine between the early β-glucan treatment group (36.0 ± 4.53) and the infected control group (41.0 ± 2.92). As regards the total larval counts in the skeletal muscles, the results are shown in Table 1. There was a significant reduction in larval counts in albendazole treatment, early β-glucan treatment, and combined treatment groups in comparison with the control group (*P* = 0.001). Moreover, a significant difference was found between larval counts in albendazole treatment and combined treatment groups (*P* = 0.001). However, no significant difference in larval counts was noted between late glucan treatment and infected control groups.

**Table 1:** Larval counts in the skeletal muscles of infected groups (*n* = 20)

	G II	G III	G IV	G V	G VI	Overall *P* value	
Mean ± SD	12,183 ± 378^a^	2906 ± 255^b^	10,905 ± 86^c^	12,400 ± 297^a^	528 ± 157^d^	< 0.0001	S
Reduction %	––––––	76.1%	10.4%	–––––-	95.6%	< 0.0001	S

G II, infected control; G III, albendazole treatment group; G IV, early β-glucan treatment group; G V, late β-glucan treatment group; G VI, combined treatment group. *S*, significant difference. Identical superscript letters denote non-significant differences while different superscript ones show statistically significant results. *P* was considered significant at < 0.05

Reduction (%) = [(*N* − *n*) / *N*] × 100, where *N* is the average number of larvae in the infected control group, and *n* is the average number of larvae in treated groups

### Assessment of immunological parameters

Table 2 shows levels of IL-5 in small intestinal homogenates and in sera of animals. Regarding IL-5 levels in intestinal homogenates, there was a significant increase in infected control mice versus uninfected controls (*P* < 0.001). On the other hand, there was a significant reduction in levels of IL-5 in both early and late β-glucan treatment groups as well as the combined treatment group in comparison with the infected control group (*P* = 0.001). Furthermore, there was a significant decrease in levels of IL-5 in both β-glucan treatment groups, compared with the albendazole treatment group (*P* = 0.001). Levels of IL-5 in sera show the same pattern as in intestinal homogenates.

**Table 2:** Comparison of the immunological parameters (*n* = 10)

	G I	G II	G III	G IV	G V	G VI	Overall *P* value	
Intestinal IL-5 (pg/gm tissue)	258.4 ± 30.8^a^	308.7 ± 14.8^b^	231.5 ± 28.2^a^	114.1 ± 21.4^c^	113.9 ± 22.8^c^	112.9 ± 16.8^c^	< 0.0001	S
Serum IL-5 (pg/ml)	62.5 ± 7.3^a^	126.2 ± 13.1^b^	126.0 ± 10.8^b^	28.2 ± 9.3^c^	31.2 ± 8.8^c^	26.3 ± 12.1^c^	< 0.0001	S
Intestinal TGF-β (pg/gm tissue)	1422.5 ± 159^a^	1850.6 ± 116.1^b^	1481.7 ± 235^a^	862.6 ± 80.9^c^	937.6 ± 65.1^c^	898.6 ± 75.0^c^	< 0.0001	S
Serum TGF-β (pg/ml)	1143.5 ± 50.9^a^	1589.6 ± 115.7^b^	1388.3 ± 210.7^c^	914.1 ± 44.6^d^	891.1 ± 79.3^d^	917.4 ± 49.3^d^	< 0.0001	S

G I, normal control; G II, infected control; G III, albendazole treatment group; G IV, early β-glucan treatment group; G V, late β-glucan treatment group; G VI, combined treatment group

Data are presented as means ± SD. *S*, significant difference. *P* value was calculated by one way ANOVA test followed by Tukey’s post-hoc test. Identical superscript letters denote non-significant differences while different superscript ones show statistically significant results. *P* was considered significant at < 0.05

As regards the levels of TGF-β in small intestinal homogenates and sera of animals, the results are shown in Table 2. There was a significant increase in infected control mice compared to normal controls (*P* = 0.001). Meanwhile, there was a significant reduction of levels of TGF-β in both β-glucan treatment groups, compared with the albendazole treatment group (*P* = 0.001). Moreover, there was a significant decrease in levels of TGF-β in both β-glucan treatment groups as well as the combined treatment group versus the infected control group (*P* < 0.001). The same pattern was observed for TGF-β levels in the sera of animals.

### Histopathological changes in the small intestine

**Fig. 1:**
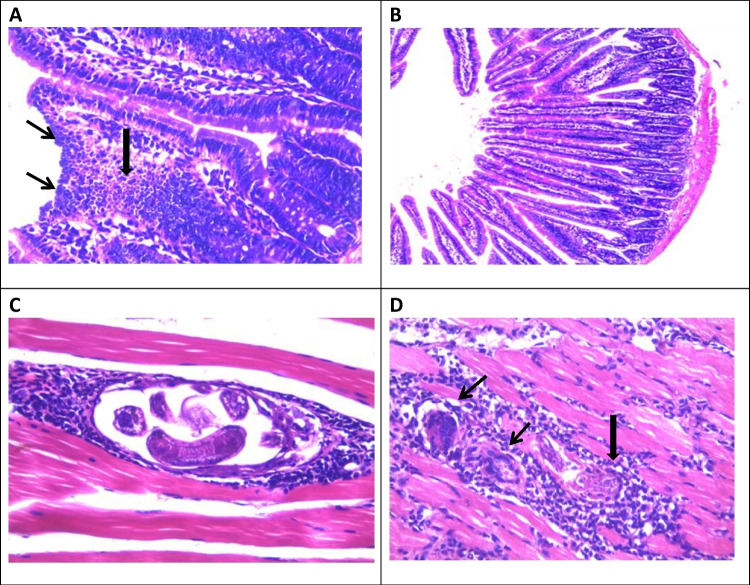
Photomicrographs of histopathological changes in *T. spiralis*-infected mice at 5 weeks p.i.: **A** a small intestinal section from the infected control group shows surface ulceration (thin arrows) and marked inflammatory cellular infiltration (thick arrow) (H&E × 400), **B** a small intestinal section from the combined treatment group shows restoration of the normal mucosal architecture (H&E × 100), **C** a muscle section from the infected control group shows intramuscular larva with intense perilarval inflammatory cellular infiltration, mainly eosinophils, macrophages and lymphocytes (H&E × 400), **D** a muscle section from the combined treatment group shows giant cells (thin arrows) with remnants of a larva (thick arrow) (H&E × 400)

Histopathological examination of sections from the infected control group showed intense inflammatory cellular infiltrate in the submucosa and the core of the villi. The infiltrate was composed mainly of plasma cells, lymphocytes, eosinophils, neutrophils, and fibroblasts. Additionally, there was ulceration of the mucosa together with goblet cell hyperplasia (Fig. 1A). The differential assessment of the components of the inflammatory cellular infiltrate is summarized in Table 3.

**Table 3:** Differential estimates of the intestinal inflammatory cellular infiltrate (*n* = 20)

	Neutrophil	Eosinophil	Histiocyte	Lymphocyte
G I	+	−	+	+ +
G II	+ + +	+ + + +	+	+ + +
G III	+ +	+ +	+ +	+ + +
G IV	+ +	+	+ + + +	+ + + +
G V	+ +	+	+ + + +	+ + + +
G VI	+ +	+	+ + + +	+ + + +

G I, normal control; G II, infected control; G III, albendazole treatment group; G IV, early β-glucan treatment group; G V, late β-glucan treatment group; G VI, combined treatment group

( −): absent; ( +): mild; (+ +): moderate; (+ + +): strong; (+ +  + +): marked

Examination of sections from either early or late β-glucan treatment groups, compared to the infected control group, revealed a mild to moderate reduction in the intensity of inflammation as well as a reduction in the number of eosinophils and an increase in the numbers of both histiocytes and lymphocytes (Table 3). Sections from the albendazole treatment group, compared to the infected control, revealed moderate improvement in the histopathological changes. Meanwhile, sections from the combined treatment group, compared to the infected control, revealed marked improvement in all the histopathological changes and the small intestine became almost normal in appearance (Fig. 1B).

**Fig. 2:**
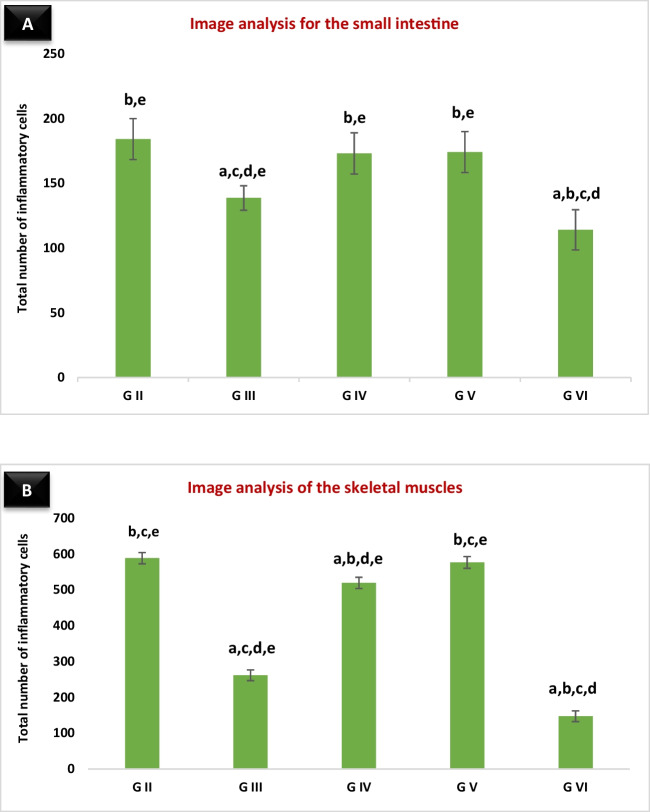
Image analysis mean values of the number of the inflammatory cells in: **A** the small intestines, and **B** the skeletal muscles of the infected groups. Vertical bars represent the mean (± SD) of these results for each group. “a” indicates a significant difference versus group II, “b” indicates a significant difference versus group III, “c” indicates a significant difference versus group IV, “d” indicates a significant difference versus group V, and “e” indicates a significant difference versus group VI. Differences were significant when *P* < 0.05

The results of the comparison between the intensities of inflammatory cellular reactions in the small intestine studied by image analysis in the different groups are illustrated in (Fig. 2A). It shows a significant reduction in the severity of inflammation in the albendazole treatment (*P* = 0.001), and the combined treatment groups (*P* < 0.001) compared with the infected control group. Furthermore, there was a significant reduction in the intensity of inflammatory infiltration in the combined treatment group compared to the albendazole treatment group (*P* < 0.05).

### Histopathological changes in the skeletal muscles

Histopathological examination of the skeletal muscle sections from the infected control group showed numerous larval depositions. The larvae were surrounded by basophilic cells with hypertrophied oval nuclei and were enclosed by collagen capsules (nurse cells). The collagen capsule was surrounded by an intense inflammatory reaction consisting of mixed cell types (histiocytes, eosinophils, lymphocytes, and plasma cells) (Fig. 1C).

Examination of sections from either early or late β-glucan treatment groups, compared to the infected control group revealed changes in the components of the inflammatory cellular infiltrate: there was a reduction in the number of acute inflammatory cells, namely eosinophils and an increase in the numbers of both histiocytes and lymphocytes. Examination of sections from the combined treatment group compared to the infected control group revealed a reduction both in the number of deposited larvae and in the inflammatory cellular infiltrate. Moreover, numerous giant cells were detected (Fig. 1D).

A comparison of the intensities of the inflammatory reactions in the skeletal muscles studied with image analysis is illustrated in Fig. 2B. We found a significant reduction in the severity of inflammatory infiltration in the albendazole treatment (*P* = 0.001) and the combined treatment (*P* < 0.001) groups compared to the infected control. Furthermore, a significant reduction in the intensity of inflammatory infiltration in the combined treatment group relative to the albendazole treatment group (*P* < 0.05) was observed. Additionally, there was a significant reduction in the severity of inflammatory reaction in the early β-glucan treatment group (*P* < 0.05), compared to the infected control group, whereas this reduction was not significant (*P* > 0.05) in the late β-glucan treatment.

### Immunohistochemical study

#### NF-κB immunostaining in the small intestine

**Fig. 3:**
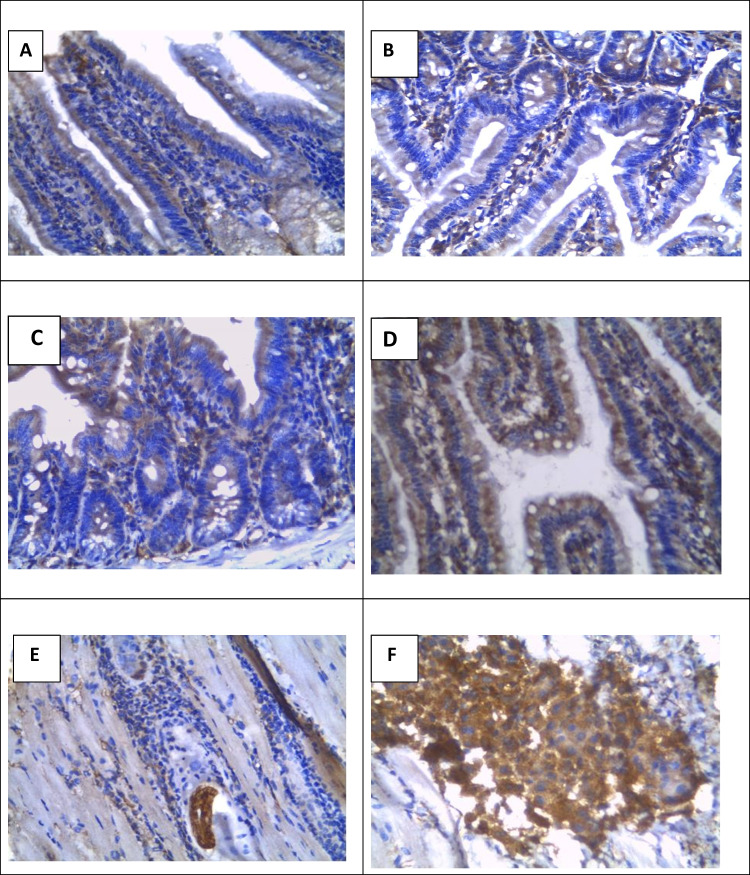
Photomicrographs of NF-κB immunostaining at 5 weeks p.i. in the small intestine (**A**‒**D**) and skeletal muscles (**E**, **F**): **A** the infected control group shows moderately positive immunoreaction for NF-κB in the nuclei and cytoplasm of enterocytes and inflammatory cells, **B** late β-glucan-treated group shows strongly positive immunoreaction in the nuclei and cytoplasm of enterocytes and inflammatory cells, **C** the albendazole group shows moderately positive immunoreaction, **D** the combined treatment group shows strongly positive immunoreaction, **E** the infected control group shows moderately positive immunoreaction for NF-κB in the nuclei and cytoplasm of inflammatory cells, **F** the combined treatment group shows strongly positive immunoreaction. (PAP × 400)

Immunohistochemical assessment of NF-κB reactivity showed a non-significant difference (*P* > 0.05) between infected control and albendazole-treated groups versus normal control. In contrast, there was a significant increase in NF-κB expression by small intestinal tissues in the early and late β-glucan treatment as well as in combined treatment groups (*P* = 0.001, *P* = 0.001, and* P* < 0.001, respectively) in comparison to the infected control group (Fig. 3A‒D; Table 4).

**Table 4:** Image analysis score of NF-κB immunostaining in tissues (*n* = 20)

	G I	G II	G III	G IV	G V	G VI	Overall *P* value	
Small intestine	81.89 ± 14.32^a^	84.94 ± 15.32^a^	83.16 ± 16.82^a^	132.17 ± 13.98^b^	133.2 ± 15.94^b^	140.6 ± 15.74^b^	< 0.0001	S
Skeletal muscles	83.14 ± 7.85^a^	85.89 ± 7.93^a^	84.40 ± 7.84^a^	120.30 ± 15.79^b^	118.26 ± 8.12^b^	116.53 ± 15.78^b^	< 0.0001	S

G I, normal control; G II, infected control; G III, albendazole treatment group; G IV, early β-glucan treatment group; G V, late β-glucan treatment group; G VI, combined treatment group

Data are presented as means ± SD. *S*, significant difference. *P* value was calculated by one way ANOVA test followed by Tukey’s post-hoc test. Identical superscript letters denote non-significant differences while different superscript ones show statistically significant results. *P* was considered significant at < 0.05

#### NF-κB immunostaining in the skeletal muscles

Immunohistochemical assessment of NF-κB reactivity in the skeletal muscle tissues showed a non-significant difference (*P* > 0.05) between infected control and albendazole-treated groups versus normal control. In contrast, there was a significantly upregulated NF-κB expression by muscle tissues in the early and late β-glucan treatment as well as in combined treatment groups (*P* = 0.001, *P* = 0.001, and *P* < 0.001, respectively) in comparison to the infected control group (Fig. 3E, F; Table 4).

## Discussion

β-glucan is considered one of the biological response modifiers. Its immune-modulating activities are linked to the capability of binding to definite receptors on the human neutrophils and macrophages. Activation of macrophages is the first line by which it enhances the host’s defense against microorganisms. Once the macrophages are activated, they have the ability to stimulate the secondary line of host defense including humoral and cellular responses. Furthermore, the release of lysosomal enzymes and leukotrienes by monocytes and the activation of the alternative complement pathway have been reported as effective host immune mechanisms induced by β-glucan (Novak and Vetvicka 2008).

Unsurprisingly, β-glucan has been widely used for enhancing host defense against infections. It showed effectiveness against both parasitic and bacterial infections including antibiotic-resistant bacteria. For example, it had been effectively used in experimental infections with *Toxoplasma gondii* (Bousquet et al. 1988), *Trypanosoma cruzi* (Williams et al. 1989), *Plasmodium berghei* (Maheshwari and Siddiqui 1989), *Leishmania major* (Goldman and Jaffe 1991), *Toxocara canis* (Hrckova et al. 2007), and *Staphylococcus aureus* (Kaiser and Kernodle 1998).

In the present work, we found that there was no significant difference in adult worm counts in the small intestine of infected control and early β-glucan treatment groups. This can be explained by the fact that β-glucan is actually not an antiparasitic drug but it has immunomodulating activities that potentiate the efficacy of some antiparasitic drugs when used as an adjuvant.

On the other hand, compared to the albendazole treatment group, there was a noticeable decrease in the number of larvae in the combined treatment group. These findings concur with those of Hrckova et al. (2007) who found that the addition of β-glucan significantly improved the effectiveness of benzimidazole carbamate anthelmintics in the treatment of dormant *Toxocara canis* larvae during late infections in mice. The potential of β-glucan to stimulate the immune system and thus increase the number of macrophages in the inflammatory cellular infiltrate around the larvae explains these outcomes. This increased phagocytic activity may result in larval injury and death. Moreover, these phagocytic cells have the ability to non-specific uptake of drugs such as albendazole. They might serve as additional drug reservoirs, slowly releasing the medication back into the body and greatly increasing its bioavailability (Ellens et al. 1982; Roerdink et al. 1984). Therefore, β-glucan appears to offer a low-cost method of enhancing the effectiveness of antiparasitic medications.

In addition, the early β-glucan treatment group had a significantly lower larval count than the infected control group, whereas the decrease was not significant in the late β-glucan treatment group in comparison with the infected control group. This could be explained by the effect of β-glucan on the mucosal immune system, which improved the function of the intestinal barrier and increased resistance to the newborn larvae. Likewise, Borosková et al. (1998) reported that β-glucan administered to animals infected with *Toxocara canis* eggs at the beginning of the infection resulted in a significant stimulation and restoration of the parasite-induced suppression of the lymphoproliferative response, and the ability of *T. canis* larvae to migrate within the tissues was reduced by about 27%.

Examination of small intestinal sections from either early or late β-glucan treatment groups compared to the infected control group revealed moderate amelioration of the severity of inflammation. These findings were confirmed by image analysis. Furthermore, sections from the combined treatment group compared to the infected control group revealed marked improvement in all the histopathological changes and the small intestine looks nearly normal. These findings might extrapolate in humans as an improvement of enteric symptoms such as diarrhea and abdominal pains as well as prevention of complications such as fluid loss, electrolyte deficiency, and bacteremia.

Interestingly, compared to the infected control group, histopathological examination of the skeletal muscle sections from β-glucan treatment groups revealed modifications in the type of cells in the inflammatory cellular infiltrate: there was a reduction in the number of eosinophils and an increase in the numbers of both macrophages and lymphocytes. These observations are in accordance with the previous findings about the immunomodulating effects of β-glucan (Kerékgyártó et al. 1996). Additionally, examination of skeletal muscle sections from the combined treatment group compared to the infected control revealed a reduction in both the number of deposited larvae and the intensity of inflammation. Notably, numerous giant cells were detected. The presence of giant cells denotes the enhanced phagocytic activity of macrophages that may help in larval killing and engulfment. These findings are in accordance with Hrckova and Velebný (2001) who concluded that β-glucan potentiates the effects of albendazole in the treatment of *Toxocara canis* infection.

Interleukin-5 (IL-5) is a key growth and differentiation agent for eosinophil granulocytes. It is not only connected to the formation and differentiation of eosinophils but may also stimulate basophils. Its effect on eosinophil production is almost immediate as the formation and survival of eosinophils almost completely stop when IL-5 expression is inhibited by medications or gene deletion (Stein and Munitz 2010). Kang et al. (2012) demonstrated that IL-5 levels were upregulated from the start of *T. spiralis* infection and remained elevated even in the chronic stage of the infection.

In the present work, there is a significant reduction in levels of IL-5 in β-glucan treatment groups compared with the infected control and albendazole treatment groups. These results are in accordance with those of Kirmaz et al. (2005) who reported similar effects of β-glucan administration in a mouse model of allergic rhinitis. These results explain the reduction in the number of eosinophils in the inflammatory cellular infiltrate whether in the gut mucosa or around the encapsulated larvae. Attenuation of the eosinophilic response of the *Trichinella*-induced hypersensitivity-mediated reactions in the intestine may again alleviate gastrointestinal manifestations and complications.

Transforming growth factor-beta is a multifunctional polypeptide hormone that influences various cell processes, such as controlling cell division, differentiation, and death; regulating immunity; controlling the inflammatory response; and promoting regrowth and healing (Fiocchi 2001). At the cellular level, TGF-β influences nearly every stage of the chronic inflammatory and fibrotic processes. The transcription of numerous extracellular matrix elements, including collagen, fibronectin, glycosaminoglycans, and metalloproteinases and their inhibitors, are regulated by TGF-β (Monteleone et al. 2001).

TGF-β is also a cytokine that is essential for controlling immune cell activity. It inhibits the proliferation of B and T lymphocytes and promotes homeostasis (Kehrl 1991). It is also involved in tissue remodeling that follows infections and injuries. Interestingly, this cytokine appears to have dual functions as it facilitates the development of Th17 and T-regulatory lymphocytes, which play vital roles in the activation and suppression of immune responses, respectively, against parasite infections (Karimi-Googheri et al. 2014). TGF-β levels in the jejunum are found to be higher during *T. spiralis* infection. This increase begins two weeks p.i., and it is accompanied by an increase in IL-17 and Th17 cells. At eight weeks p.i., its levels then return to normal (Fu et al. 2009). Furthermore, Beiting et al. (2007) proved that the combined deficiency of TGF-β and IL-10 was associated with the death of encapsulated muscle larvae. Thus, TGF-β seems to synergize with IL-10 in the control of local inflammation, and their lack leads to a more severe inflammatory response in muscles.

In the present work, there was a significant increase in levels of TGF-β in small intestinal homogenates and in sera of infected control mice compared to uninfected controls. On the other hand, there was a significant decrease in levels of TGF-β in β-glucan treatment groups compared with the infected control and albendazole treatment groups. This downregulation of the levels of TGF-β could be considered beneficial in the treatment of trichinellosis as it may help mitigate the inflammatory response in the intestine and induce parasite demise in the skeletal muscles.

In an attempt to explore one of the pathways of action of β-glucan and to explain, at least partly, its immunomodulating potential, an immunohistochemical assessment by NF-κB immunoreactivity was done. NF-κB is a transcription factor that controls various processes such as inflammation, wound healing, stress response, apoptosis, and angiogenesis. Additionally, it is a key transcription factor regulating genes implicated in T-cell development, maturation, and proliferation (Livolsi et al. 2001). It is evidently stimulated in inflamed intestinal tissues, particularly in macrophages and epithelial cells. The degree of its activation correlates with the degree of bowel inflammation (Ashour et al. 2015).

In this work, there was a significant increase in NF-κB expression by small intestinal tissues and skeletal muscle tissues in the β-glucan treatment groups in comparison to other groups. These results are similar to those of Volman et al. (2010) who found that β-glucan-treated mice showed an increased intestinal NF-κB transactivation in leukocytes and enterocytes, particularly in the proximal part of the small intestine.

According to some in vitro experiments, excretory‒secretory antigens from *T. spiralis* strongly suppress NF-κB in activated macrophages (Bai et al. 2012). On the other hand, it was shown that NF-κB activation aided in infection eradication by triggering Th2 cytokine responses (mostly IL-9 and IL-13) (Else et al. 1994; Helmby et al. 2001). Our results showed that there was upregulated expression of NF-κB in small intestinal and skeletal muscle tissues in β-glucan-treated mice, and this may explain the enhancement of some components of the innate system, e.g., the macrophages and neutrophils. Moreover, activation of NF-κB in the skeletal muscles may affect the process of nurse cell formation via its effects on inflammation, apoptosis, and angiogenesis—a notion that needs further evaluation. Thus, NF-κB activation may be one of the pathways through which β-glucan exerts its functions.

Interestingly, there is an association between TGF-β and NF-κB; TGF-β usually suppresses NF-κB activity in normal cells, whereas NF-κB activation induces Smad7 expression, which sequentially inhibits TGF-β signaling via Smads (proteins inside cells that transmit TGF-ligand extracellular signals to the nucleus, where they stimulate downstream gene transcription) (Hong et al. 2007; Lee et al. 2010). Our results were in agreement with these studies as we found that under the effect of β-glucan treatment, there was a significant increase in NF-κB expression and a significant decrease in TGF-β levels. Other possible mechanisms of action of β-glucan have to be explored in further research.

Interestingly, two recent studies pointed out the protective role of β-glucans against experimental *T. spiralis* infection. Liu et al. (2021) showed that β-glucan can be considered a promising adjuvant when combined with *T. spiralis* recombinant antigen in a vaccine. β-glucan enhanced the immune effector mechanisms such as antibody production and Th1/Th2 cytokine production in immunized mice. In another context, Jin et al. (2022) found that β-glucan facilitated worm expulsion during *T. spiralis* infection in mice and attributed the protective effect to the expansion of gut microbiota, particularly *Akkermansia muciniphila*. The latter reinforced the function of the intestinal mucus layer via interaction with toll-like receptor 2. The results of these studies may be relevant to the data observed in our study in one way or another.

In conclusion, β-glucan can be considered an effective adjuvant in the treatment of trichinellosis. Combined with albendazole, β-glucan was able to mitigate the inflammatory reactions in the small intestine and skeletal muscles and to improve the immunological parameters. Surprisingly, it potentiated the antiparasitic activity of albendazole as evidenced by the reduction of muscle larval burden in combined treatment. Overall, our data indicate the beneficial multifaceted impact of β-glucan on the therapeutic response during experimental trichinellosis. Therefore, human studies are worthwhile to determine the best therapeutic strategy combining both antiparasitic agents and β-glucan.

## Data Availability

The datasets generated during the current study are available from the corresponding author on reasonable request.
